# Diabetic Embryopathy Susceptibility in Mice Is Associated with Differential Dependence on Glucosamine and Modulation of High Glucose-Induced Oxidative Stress

**DOI:** 10.3390/antiox10081156

**Published:** 2021-07-21

**Authors:** Jin Hyuk Jung, Mary R. Loeken

**Affiliations:** Section on Islet Cell and Regenerative Biology, Joslin Diabetes Center, Department of Medicine, Harvard Medical School, Boston, MA 02215, USA; jungjh@skinresearch.or.kr

**Keywords:** diabetic pregnancy, hexosamine biosynthesis, glucose transport, embryo metabolism

## Abstract

The high K_M_ glucose transporter, GLUT2 (SLC2A2), is expressed by embryos and causes high rates of glucose transport during maternal hyperglycemic episodes in diabetic pregnancies and causes congenital malformations (diabetic embryopathy). GLUT2 is also a low K_M_ transporter of the amino sugar, glucosamine (GlcN), which enters the hexosamine biosynthetic pathway (HBP) and provides substrate for glycosylation reactions. Exogenous GlcN also increases activity of the pentose phosphate pathway (PPP), which increases production of NADPH reducing equivalents. GLUT2-transported GlcN is inhibited by high glucose concentrations. Not all mouse strains are susceptible to diabetic embryopathy. The aim of this study was to test the hypothesis that susceptibility to diabetic embryopathy is related to differential dependence on exogenous GlcN for glycosylation or stimulation of the PPP. We tested this using murine embryonic stem cell (ESC) lines that were derived from embryopathy-susceptible FVB/NJ (FVB), and embryopathy-resistant C57Bl/6J (B6), embryos in the presence of low or high glucose, and in the presence or absence of GlcN. There were no significant differences in *Glut2* expression, or of glucose or GlcN transport, between FVB and B6 ESC. GlcN effects on growth and incorporation into glycoproteins indicated that FVB ESC are more dependent on exogenous GlcN than are B6 ESC. GlcN stimulated PPP activity in FVB but not in B6 ESC. High glucose induced oxidative stress in FVB ESC but not in B6 ESC. These results indicate that FVB embryos are more dependent on exogenous GlcN for glycosylation, but also for stimulation of the PPP and NADPH production, than are B6 embryos, thereby rendering FVB embryos more susceptible to high glucose to induce oxidative stress.

## 1. Introduction

Maternal diabetes that is present prior to pregnancy (pregestational diabetes) significantly increases risk for congenital malformations, a diabetic complication known as “diabetic embryopathy”; in both human fetuses and in animal models, neural tube defects NTDs are among the most common that occur [1,2,3,4]. Using a mouse model of diabetic pregnancy, we have shown that maternal hyperglycemia, through high rates of glucose transport into embryo cells via the high K_M_ glucose transporter, GLUT2 (SLC2A2), is responsible for diabetes-induced NTDs [5,6]. Several studies using animal models have shown that maternal diabetes-induced oxidative stress is central to diabetic embryopathy [7,8,9,10,11,12]. Several studies from our lab have shown that high glucose-induced oxidative stress inhibits gene expression that is required for neural tube closure and inhibition of apoptosis, thereby causing NTDs [6,12,13,14].

GLUT2 is not likely to function as a physiological glucose transporter for the embryo when mothers are not hyperglycemic, because the GLUT2 K_M_ for glucose (approximately 16 mmol/L) is significantly higher than normal blood glucose concentrations (5.5 mmol/L) and embryos also express the low K_M_ (approximately 5.5 mmol/L) glucose transporters, GLUT1 and GLUT3, which would transport glucose more efficiently than GLUT2 during euglycemia [15,16]. We noticed that *Glut2*-deficient embryos have a survival disadvantage compared to their wild type littermates [5], suggesting that GLUT2 serves an important survival function during normal embryogenesis. GLUT2 is also a low K_M_ (0.8 mmol/L) glucosamine (GlcN) transporter [17]. We recently showed that GlcN stimulates proliferation of embryonic stem cells (ESC) in a GLUT2-dependent manner [18]. Upon transport and phosphorylation, GlcN enters the hexosamine biosynthetic pathway (HBP), which provides substrates for glycosylation reactions. GlcN-6-PO_4_ can also be synthesized from fructose-6-PO_4_ + glutamine; however, uptake of exogenous GlcN increases substrates for glycosylation, while decreasing entry of fructose-6-PO_4_ and glutamine into the HBP, thereby increasing substrates for the pentose phosphate pathway (PPP), glycolysis, and the tricarboxylic acid (TCA) cycle [18]. GLUT2-transported glucose inhibits uptake of GlcN [18]. Therefore, some of the adverse effects of maternal hyperglycemia to induce malformations might be not only due to increased glucose uptake and metabolism, but also to insufficient GlcN uptake and metabolism.

We previously reported that C57Bl/6J (B6) embryos, unlike FVB/NJ (FVB) embryos, are resistant to NTDs induced during diabetic pregnancy, despite similar levels of maternal hyperglycemia; this is correlated with failure to inhibit expression of *Pax3*, which is required for neural tube closure, in B6 embryos of diabetic mothers [19]. Thus, strain background differences may be useful to reveal metabolic pathways that are essential for diabetic embryopathy. Because GLUT2 confers susceptibility to hyperglycemia-induced NTDs [5], and high glucose concentrations significantly inhibit GLUT2-transported GlcN [18], we hypothesized that the differences in susceptibility to diabetic embryopathy of FVB and B6 embryos is due to differential dependence on exogenous (maternal) GlcN to support hexosamine biosynthesis, promote growth, and regulate oxidative stress. Thus, when embryos are exposed to elevated glucose concentrations, decreased GlcN transport could adversely affect development of FVB, but not in B6, embryos.

This hypothesis cannot be tested in vivo because, while maternal GlcN levels can be raised, they cannot be lowered or eliminated. Instead, here we tested whether differential GlcN metabolism is correlated with susceptibility to diabetic embryopathy, using ESC that were derived from FVB or B6 blastocysts in low glucose media (LG-ESC) that retain expression of functional GLUT2 transporters [20,21]. We first tested whether FVB and B6 embryos exhibit differential susceptibility to NTDs specifically in response to maternal hyperglycemia, as they do in response to maternal diabetes, which is necessary in order to validate the use of LG-ESC derived from these strains to study effects of glucose and GlcN on metabolic pathways associated with diabetic embryopathy. We next compared expression of *Glut2* by the two strains, and studied transport of the HBP substrates, glucose, glutamine, and GlcN, as well as utilization of glutamine or GlcN for incorporation into glycoproteins. Finally, we tested the effects of GlcN on growth of pluripotent ESC colonies by the two cell lines, as well as activity of the PPP and markers of oxidative stress.

## 2. Materials and Methods

### 2.1. Animals

All animal procedures were approved by the Institutional Animal Care and Use Committee (IACUC) of the Joslin Diabetes Center. Six-week-old female and male FVB and B6 mice were purchased from the Jackson Laboratory (Bar Harbor, ME, USA) and were maintained on a 12-h light–dark cycle with free access to food (PicoLab Mouse Diet 20) and water. Nondiabetic female mice were housed with nondiabetic males of the same strain and were checked daily for copulation plugs. Noon on the day that a copulation plug was found was determined to be embryonic day (E) 0.5. To induce hyperglycemia (>16.65 mmol/L) during a 9-h period on E7.5, which has previously been shown to replicate the embryopathic effects of diabetic pregnancy, pregnant mice were injected with 2 mL of 12.5% glucose in phosphate buffered saline (PBS) on E7.5 at approximately hourly intervals from 9:00 a.m.–5:00 p.m. as described [6]. Embryos were dissected from uteri on E7.5 or 10.5.

### 2.2. Culture of FVB and B6 LG-ESC

Murine ESC that were isolated in low glucose (5.5 mmol/L) Dulbecco’s modified Eagle medium (DMEM) from FVB and B6 blastocysts (FVB LG-ESC and B6 LG-ESC) [20,21] were grown in the absence of a feeder layer on culture dishes coated with 0.1% gelatin, in incubators containing 5% O_2_ and 5% CO_2_ (balance N_2_) as described [18,21]. ESC were induced to differentiate into neuronal precursors as previously described [21,22], using either low or high glucose (17.5 mmol/L) media during selection of neuronal precursors from embryoid bodies [21]. All cultures were performed in triplicate.

### 2.3. RT-PCR Assays

Total RNA was extracted using TRIzol reagent (Thermo Fisher Scientific, Waltham, MA, USA). Two hundred ng RNA were reverse transcribed in quadruplicate using the High-Capacity cDNA Reverse Transcription Kit (Thermo Fisher Scientific). Real-time PCR was performed using TaqMan PCR Master Mix (Thermo Fisher Scientific) and primers and VIC-labeled probe to detect 18S *rRNA* (Thermo Fisher Scientific) as the normalization control as described [12]. Primers and FAM-labeled probe for *Pax3* cDNA were previously reported [12]. Primers and FAM-labeled probe for *Glut2* cDNA were obtained from Thermo Fisher Scientific.

### 2.4. Immunoblot Assays

Thirty micrograms of whole cell protein extracts were analyzed by immunoblot as described [23]. Sources and dilutions of primary and secondary antibodies used to detect GLUT1, GLUT2, GLUT3, and β-ACTIN were as reported previously [18]. The glutamine transporters ASCT2, LAT1, SNAT1, and SNAT5 were detected with rabbit anti-ASCT2/SLC1A5 (1:500, Abcam, Cambridge, MA, USA), rabbit anti-LAT1 (1:1000, Santa Cruz Biotechnology, Dallas, TX, USA), rabbit anti-SNAT1/SLC38A1 (1:200, Abcam), and goat anti-SNAT5/SLC38A5 (1:100, Santa Cruz Biotechnology), respectively. Secondary antibodies were as reported previously [18] or donkey anti-goat IgG HRP-coupled (1:3000, Santa Cruz Biotechnology). Secondary antibodies were detected by Western Lightning *Plus*-ECL (Thermo Fisher Scientific) and exposure to X-ray film.

### 2.5. Alkaline Phosphatase Staining

Cells were cultured for 4 days, then colonies were stained for alkaline phosphatase (AP) using Fast Red TR salt and Naphthol (both from Sigma-Aldrich, St. Louis, MO, USA) as described [18] and then AP+ colonies in culture dishes were counted.

### 2.6. Glucosamine, 2-Deoxy-d-Glucose, and Glutamine Transport Assays

Transport of ^3^H-GlcN and the fluorescent 2-deoxy-D-glucose analog, 2-NBDG, by FVB and B6 LG-ESC were measured as described [17,18,21]. Transport of ^3^H-glutamine was measured as described for ^3^H-GlcN transport [17,18] except that cells were grown for 2 days in glutamine-free media supplemented with 200 μmol/L or 4 mmol/L glutamine + 0.8 mmol/L GlcN. Cells were incubated in buffer containing 5 mmol/L 2-deoxy-d-glucose, 200 μmol/L or 4 mmol/L glutamine containing 0.5 or 10 μCi 3H-glutamine, respectively, +0.8 mmol/L GlcN, for 20 min.

### 2.7. Glycoprotein Detection

Total N-glycosylated proteins were detected by electrophoresis of 10 μg cell extract on a 10% polyacrylamide gel, followed by periodic acid-Schiff (PAS) staining using a Pierce^TM^ Glycoprotein Staining kit (Thermo Scientific, Waltham, MA, USA). Gels were scanned and bands were quantified using Adobe Photoshop CS5.1 software. Total *O*-GlcNAcylated proteins were identified by immunoblot as described [13]. Incorporation of glutamine or GlcN into total *O*- and *N*-glycosylated proteins was assayed by metabolic labeling of wheat germ agglutinin (WGA)-precipitable protein with ^3^H-glutamine or ^3^H-GlcN. Briefly, ^3^H-glutamine labeling was performed by culturing cells for 2 days using low glucose DMEM (which contains 4 mmol/L glutamine) + 0.8 mmol/L GlcN, then media were replaced with the same media containing 2 μCi/mL ^3^H-glutamine and cultured for 2 additional days. ^3^H-GlcN labeling was performed by culturing cells in low glucose media (which contains 4 mmol/L glutamine) for 2 days, then media was replaced with media containing 0.8 mmol/L GlcN + 2 μCi/mL 3H-GlcN and cultured for 2 additional days. At the end of culture, cells were washed with cold PBS, scraped to remove them from culture dishes, sonicated, and then glycoproteins were isolated by adsorption to WGA-agarose beads (Thermo Scientific, Waltham, MA, USA) following the manufacturer’s instructions.

### 2.8. Glucose-6-PO_4_ Dehydrogenase (G6PD) Assay

G6PD activity was assayed after 48 h of culture + 0.8 mmol/L GlcN as described [18,24,25].

### 2.9. Malondialdehyde Assay

Cells were cultured in low glucose media. After 40 h, media were replaced with low or high glucose media and cultured for an additional 8 h. Malondialdehyde was assayed by thiobarbituric acid reactivity as described [21].

### 2.10. Statistical Analyses

Data were analyzed by two-tailed Student’s t-test, by one- or two-way ANOVA followed by Tukey’s multiple comparisons test, using GraphPad Prism software v. 8 (San Diego, CA, USA). A *p* value < 0.05 was used as statistically significant.

## 3. Results

### 3.1. Differential Susceptibility of FVB and B6 Embryos and LG-ESC to the Adverse Effects of High Glucose

**Figure 1:**
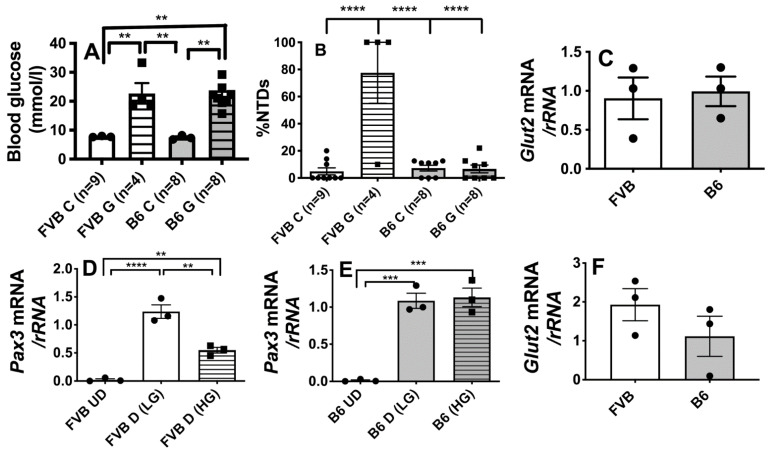
Differential effects of maternal hyperglycemia or high glucose culture on FVB/NJ (FVB) and C57Bl/6J (B6) embryos and ESC. (**A**) Average hourly blood glucose levels of pregnant control (C) or transiently hyperglycemic from glucose injection (G) FVB or B6 dams on embryonic day (E) E7.5 (*n* = 4–9 litters/treatment group). (**B**) Percent of embryos with neural tube defects (NTDs) per litter on E10.5 from control or hyperglycemic dams shown in A. (**C**) *Glut2* expression by E7.5 FVB or B6 embryos (*n* = 3 litters each) as determined by real-time RT-PCR of *Glut2* mRNA normalized to *rRNA*. (**D**) *Pax3* expression by undifferentiated (UD) or differentiating neuronal precursors (D) FVB LG-ESC cultured in low (5.5 mmol/L) or high (17.5 mmol/L) glucose media during selection of neuronal precursors as determined by real-time RT-PCR (*n* = 3 replicate culture dishes). (**E**) *Pax3* expression by B6 LG-ESC cultured and analyzed as in panel D. (**F**) *Glut2* expression by undifferentiated FVB or B6 LG-ESC as determined by real-time RT-PCR. Data shown are means ± SEM. Data in panels A, B, D, and E were analyzed by one-way ANOVA followed by Tukey’s multiple comparisons test. Data in panels C and F were analyzed by Student’s t-test. ** *p* < 0.01; *** *p* < 0.001; **** *p* < 0.0001.

We previously showed that transient maternal hyperglycemia (≥14 mmol/L) on E7.5 is necessary and sufficient to induce NTDs in embryos of mouse strains that are susceptible to diabetic embryopathy [6]. In order to validate the use of LG-ESC derived from mouse strains that differ in susceptibility to diabetic embryopathy as a model to understand whether this differential embryopathy susceptibility is related to GlcN uptake or metabolism, we first tested whether B6 embryos, which are resistant to NTDs induced by diabetic pregnancy, were resistant to NTDs specifically induced by maternal hyperglycemia in the absence of other metabolic disturbances associated with diabetes. Mean blood glucose levels were significantly and similarly increased by glucose injections over a 9-h period on E7.5 (>21 mmol/L) in both FVB and B6 dams (Figure 1A). However, maternal hyperglycemia significantly increased NTDs only in FVB embryos (Figure 1B). There were no differences in expression of Glut2 mRNA during induction of the neural tube (E7.5, Figure 1C) between FVB or B6 embryos that would explain differences in susceptibiltiy of FVB and B6 embryos to hyperglycemia-induced NTDs.

We next tested whether LG-ESC derived from FVB and B6 blastocysts cultured in high glucose media replicate the differential susceptibility of embryos of these strains to inhibition of *Pax3* expression, which occurs in response to maternal diabetes and hyperglycemia and is associated with NTDs in FVB, but not B6 embryos [19]. Expression of *Pax3* was induced upon differentiation of FVB and B6 LG-ESC to neuronal precursors, but high glucose significantly inhibited *Pax3* expression only by FVB LG-ESC-derived neuronal precursors (Figure 1D,E). There was no significant difference in *Glut2* expression by FVB or B6 LG-ESC (Figure 1F). The differential susceptibility of embryos of these strains to the inhibitory effects of high glucose culture on *Pax3* expression, and lack of differences in *Glut2* expression, indicate that FVB and B6 LG-ESC can be used to study whether the strain differences in susceptibility to diabetic embryopathy are related to differences in GlcN transport or metabolism.

### 3.2. Hexosamine Substrate Transport by FVB and B6 LG-ESC

**Figure 2:**
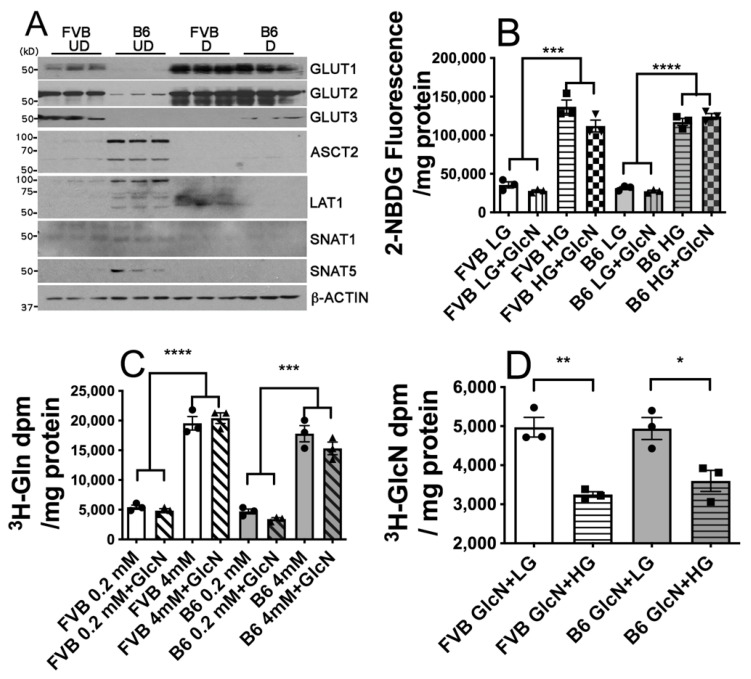
Transport of the hexosamine biosynthetic pathway substrates glucose, glutamine, and GlcN by FVB and B6 LG-ESC. (**A**) Immunoblot analysis of steady state levels of the glucose transporters, GLUT1 (~55 kDa), GLUT2 (~57 kDa), and GLUT3 (~47 kDa), and the glutamine transporters, ASCT2 (~56 kDa), LAT1 (~55 kDa), SNAT1 (~48 kDa), and SNAT5 (~52 kDa), and β-ACTIN (~42 kDa) as a loading control, from three replicate culture dishes of undifferentiated (UD) or differentiating neuronal precursors (D) from FVB or B6 LG-ESC. (**B**) Transport of 5 (LG) or 16 mmol/L (HG) fluorescent 2-deoxy-D-glucose (2-NBDG) in the presence or absence of 0.8 mmol/L GlcN. (**C**) Transport of 0.2 or 4 mmol/L ^3^H-glutamine (Gln) in the presence or absence of 0.8 mmol/L GlcN. (**D**) Transport of 0.8 mmol/L ^3^H-GlcN in the presence or absence of 5 (LG) or 16 (HG) mmol/L glucose. Data shown are means ± SEM and were analyzed by two-way ANOVA followed by Tukey’s multiple comparisons test (**B**,**C**) or Student t test (**D**). * *p* < 0.05; ** *p* < 0.01; *** *p* < 0.001; **** *p* < 0.0001.

If the differential responsiveness of FVB and B6 embryos and LG-ESC to the effects of high glucose is related to differential reliance on exogenous GlcN, this suggests that strains that are less dependent on exogenous GlcN, and less sensitive to the adverse effects of high glucose, would be more dependent on glucose (at physiological concentrations via low K_M_ transporters) and glutamine to provide substrates for hexosamine biosynthesis, as well as for growth and energy production. If this is the case, there may be differences in steady state levels of low and high K_M_ glucose transporters (i.e., high and low K_M_ GlcN transporters) and glutamine transporters, and/or functional transport of glucose, glutamine, and GlcN of FVB and B6 LG-ESC that indicate differential substrate utilization. Immunoblots demonstrated that there were several differences in steady state protein levels, both between LG-ESC of either strain at the same stage of differentiation, and between undifferentiated and differentiating neuronal precursors of each strain, of the glucose transporters, GLUT1, GLUT2, and GLUT3, and the glutamine transporters, ASCT2, LAT1, SNAT1, and SNAT5 (Figure 2A). However, the patterns of these differences in protein levels of glucose or glutamine transporters did not explain differential reliance on uptake of GlcN versus uptake of glucose + glutamine between the strains, or at different stages of differentiation.

To investigate whether there are functional differences in the transport of substrates for the hexosamine biosynthetic pathway in FVB and B6 LG-ESC, we assayed uptake of the fluorescent 2-deoxy-D-glucose analog, 2-NBDG (using 5 or 16 mmol/L to distinguish uptake by low and high K_M_ glucose transporters) in the presence or absence of 0.8 mmol/L GlcN, uptake of ^3^H-glutamine (using 0.2 or 4 mmol/L to distinguish uptake by low and high K_M_ glutamine transporters [26,27,28]) in the presence or absence of 0.8 mmol/L GlcN, or uptake of ^3^H-GlcN in the presence of 5 or 16 mmol/L glucose. Transport of 16 mmol/L 2-NBDG was significantly increased compared to 5 mmol/L 2-NBDG by both FVB and B6 LG-ESC (Figure 2B), suggesting that both cell lines express functional GLUT2 transporters. There was no significant inhibition of either 5 or 16 mmol/L 2-NBDG uptake by 0.8 mmol/L GlcN by either cell line, suggesting that availability of exogenous GlcN does not affect transport of glucose by either low or high K_M_ glucose transporters. Transport of 4 mmol/L ^3^H-glutamine was significantly increased compared to 0.2 mmol/L ^3^H-glutamine by both cell lines, and there was no effect of GlcN on ^3^H-glutamine uptake by either cell line (Figure 2C), suggesting that availability of exogenous GlcN does not affect transport of glutamine by either low- or high-K_M_ glutamine transporters. Both FVB and B6 LG-ESC transported ^3^H-GlcN, and transport of ^3^H-GlcN was significantly inhibited by 16 mmol/L glucose in both cell lines (Figure 2D). This indicated that GLUT2 functions as a GlcN transporter for both FVB and B6 LG-ESC, and that high glucose concentrations compete with GlcN for GLUT2-mediated uptake. Nevertheless, there were no functional differences in transport of any of the substrates for the hexosamine biosynthetic pathway by the two cell lines.

### 3.3. Protein Glycosylation Substrate Utilization in FVB and B6 LG-ESC

**Figure 3:**
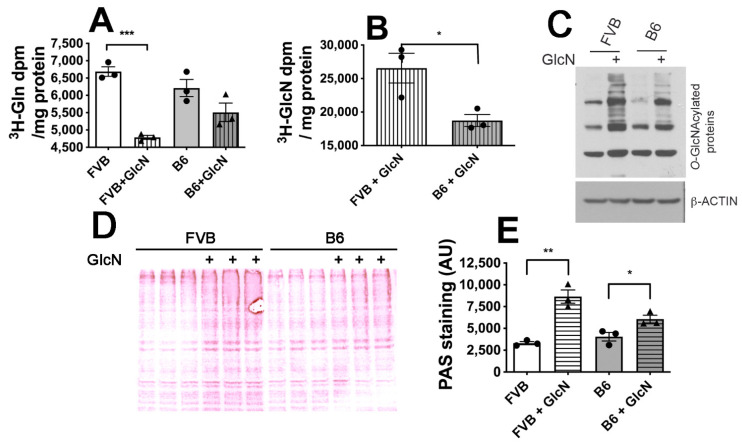
Glycosylation substrate utilization by FVB and B6 LG-ESC. (**A**) WGA-precipitated glycoproteins labeled with ^3^H-glutamine from FVB and B6 LG-ESC cultured ± 0.8 mmol/L GlcN. (**B**) WGA-precipitated glycoproteins labeled with ^3^H-GlcN from FVB and B6 LG-ESC. (**C**) Immunoblot of *O*-GlcNAcylated proteins following culture of FVB or B6 LG-ESC ± 0.8 mmol/L GlcN. (**D**) Periodic acid-Schiff (PAS) stain of *N*-glycosylated proteins separated by SDS-Polyacrylamide gel electrophoresis from three replicate culture dishes following culture of FVB or B6 LG-ESC ± 0.8 mmol/L GlcN. (**E**) Quantitation of PAS staining from (**D**). Data shown are means ± SEM and were analyzed by Student t test. * *p* < 0.05; ** *p* < 0.01; *** *p* < 0.001.

We next tested whether utilization of glucose, glutamine, and GlcN as substrates for hexosamine biosynthesis by FVB and B6 LG-ESC may differ by assaying the incorporation of ^3^H-glutamine or ^3^H-GlcN into (total *N*- and *O*-glycosylated) glycoproteins. GlcN significantly inhibited ^3^H-glutamine incorporation by FVB, but not by B6, LG-ESC (Figure 3A). There was significantly less ^3^H-GlcN incorporation by B6 LG-ESC than by FVB LG-ESC (Figure 3B). We further tested whether there were differences in levels of *O*- or *N*-glycosylation when only glucose + glutamine were available as substrates, or when glucose, glutamine, and GlcN were available. Steady state levels of *O*-GlcNAcylated proteins were assayed by immunoblot and were increased by supplemental GlcN in both cell lines (Figure 3C). Levels of *N*-glycosylated proteins were assayed by periodic acid-Schiff (PAS) staining of SDS-polyacrylamide gels and were significantly increased by GlcN in both cell lines; however, the increased glycosylation in FVB LG-ESC was 2.6-fold, and in B6 LG-ESC was only 1.4-fold (Figure 3D,E).

### 3.4. Growth Metabolic Effects of GlcN Uptake in FVB and B6 LG-ESC

We previously showed that GLUT2-transported GlcN stimulates growth and proliferation of FVB LG-ESC, which appears to result from decreased flux of fructose-6-PO_4_ + glutamine into the HBP, thereby increasing levels of glycolytic intermediates and glutamine that can be used for biomass accumulation and for maintaining energy production [18]. With decreased flux of fructose-6-PO_4_ into the HBP, there is increased accumulation of glucose-6-PO_4_, which stimulates activity of the PPP [18], thereby increasing substrates for nucleotide synthesis, as well as NADPH to increase reduction of the antioxidant, reduced glutathione (GSH) from oxidized glutathione (GSSG).

**Figure 4:**
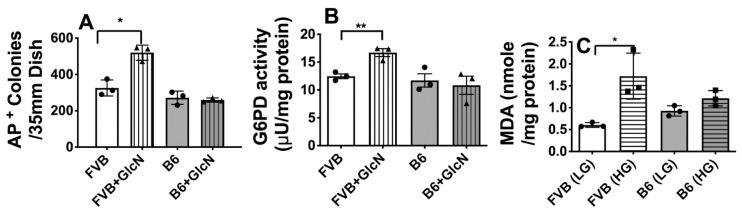
Relationships between differences in GlcN utilization on GlcN stimulation of growth, pentose phosphate pathway (PPP) activities, and high glucose-induced oxidative stress between FVB and B6 LG-ESC. (**A**) Alkaline phosphatase-positive (AP+) colonies following culture in low glucose media ± 0.8 mmol/L GlcN. (**B**) G6PD enzyme activity by FVB and B6 LG-ESC cultured ± 0.8 mmol/L GlcN. (**C**) Intracellular malondialdehyde (MDA) concentrations in FVB or B6 LG-ESC cultured for 8 h in low or high glucose media. Data shown are means ± SEM and were analyzed by Student t test (B) * *p* < 0.05; ** *p* < 0.01.

To test whether B6 LG-ESC and embryos are less dependent on GlcN for stimulation of growth and the PPP than FVB LG-ESC and embryos, we cultured FVB or B6 LG-ESC with or without GlcN and counted numbers of alkaline phosphatase-positive (i.e., pluripotent) colonies, and assayed (glucose-6-phosphate dehydrogenase) G6PD activity, the rate-controlling enzyme of the PPP. GlcN significantly increased numbers of alkaline phosphatase-positive FVB LG-ESC colonies, as observed previously [18], but there was no effect of GlcN on B6 LG-ESC colonies (Figure 4A). Notably, GlcN significantly stimulated G6PD activity in FVG LG-ESC, but had no effect on G6PD activity in B6 LG-ESC (Figure 4B). As a consequence of stimulation of the PPP by GLUT2-transported GlcN in FVB, but not B6, embryo cells, we considered that there may be increased oxidative stress in response to high glucose in FVB, but not B6 embryo cells. In support of this, high glucose media increased levels of malondialdehyde, a marker of oxidative stress, by FVB LG-ESC, but not by B6 LG-ESC (Figure 4C).

## 4. Discussion

We previously showed that B6 embryos are resistant to diabetic embryopathy, compared to FVB embryos [19]. The data presented here show that B6 embryos are specifically resistant to maternal hyperglycemia just prior to induction of neural tube formation, independent of other metabolic disturbances of diabetes, and this is not due to differences in expression of *Glut2* mRNA, which encodes the high K_M_ glucose transporter that is required for susceptibility to hyperglycemia-induced NTDs [5]. High glucose culture inhibited expression of *Pax3*, a gene whose inhibition leads to diabetic pregnancy-induced NTDs [13,19], by LG-ESC derived from FVB, but not B6 embryos, even though expression of *Glut2* by these cell lines does not differ. This demonstrated that FVB and B6 LG-ESC can be used to study the differential susceptibility of these strains to the adverse embryonic effects of high glucose. An in vitro model is essential to investigate whether the differential susceptibility of these strains is related to GlcN transport or metabolism because it is not possible to eliminate or lower maternal GlcN concentrations, and efforts to raise maternal GlcN by supplemental administration would increase high K_M_ GlcN transport by GLUT1 [17].

There were no differences between GlcN transport by FVB or B6 LG-ESC, or of the other substrates that can be used for hexosamine biosynthesis, glucose, and glutamine. As observed previously for FVB LG-ESC [18], GLUT2-transported glucose significantly inhibited uptake of GlcN by both cell lines. While this supports the possibility that the adverse effects of high glucose might not just be due to increased glucose uptake, but also due to decreased GlcN uptake, it does not explain the insensitivity of B6 embryo and ESC to the adverse effects of high glucose. However, differences in ^3^H-glutamine and ^3^H-GlcN incorporation into WGA-precipitable protein suggests that FVB LG-ESC utilize exogenous GlcN over fructose-6-PO_4_ + glutamine for glycosylations compared to B6 LG-ESC. By utilizing exogenous GlcN for the HPB, glycolytic intermediates and glutamine in FVB LG-ESC are spared and can be used for biomass accumulation and stimulation of the PPP. Thus, when there is increased GLUT2-transported glucose (and less GLUT2-transported GlcN) by FVB LG-ESC, there is less generation of NADPH, leading to increased oxidative stress. We previously suggested that GlcN may be an essential nutrient for embryos because they cannot synthesize sufficient amounts of HBS substrate from fructose-6-PO_4_ + glutamine without compromising biomass accumulation [18]. Our data suggest that GlcN may not be essential for all mouse strains. While *O*- and *N*-glycosylations were increased by GlcN in B6 LG-ESC, growth, and substrates derived from the PPP, were not inhibited in B6 LG-ESC in the absence of exogenous GlcN.

Of note, if B6 embryos and ESC are more reliant on fructose-6-PO_4_ + glutamine, than on GlcN, for GlcN-6-PO_4_ production, this would cause increased glutamate production, which may lead to increased GSH synthesis [29]. This could further protect B6 embryos and ESC from high glucose-induced oxidative stress.

There have been some reports that stimulation of *O*-GlcNAc transferase, which catalyzes *O*-linked glycosylation of serine and threonine residues with UDP-N-Acetyl-GlcN, by GlcN promotes proliferation of murine ESC [30,31,32]. There was also a recent report that proliferation of mouse embryo fibroblasts (MEFs) requires noncatalytic functions of *O*-GlcNAc transferase [33]. However, the ESC and MEF lines used in those studies were isolated under conventional high glucose conditions and would not express GLUT2, and the ESC were treated with GlcN at concentrations that would be transported by GLUT1 [17]. Using our FVB LG-ESC, we found that there is no effect of GlcN to stimulate pluripotency or to inhibit differentiation, and inhibiting *O*-GlcNAc transferase activity or knocking down its expression with shRNA showed that stimulation of proliferation by GlcN is not due to increased *O*-GlcNAc transferase activity [18]. Rather, stimulation of proliferation by GlcN appears to be due to increased availability of glycolytic intermediates and glutamine for biomass accumulation [18].

Kim, et al. reported that an *O*-GlcNAc transferase inhibitor reduced NTDs and alleviated oxidative stress in embryos of diabetic mice, suggesting that increased *O*-linked glycosylation in response to maternal hyperglycemia contributes to diabetic embryopathy [34]. However, we showed that NTDs induced by maternal hyperglycemia or administration of GlcN (at concentrations that would be transported by GLUT1) were prevented by supplemental GSH [24]. It is possible that the effect *O*-GlcNAc transferase inhibition in embryos of diabetic mice by Kim, et al. caused HBP intermediates upstream of UDP-N-Acetyl-GlcN, including GlcN-6-PO_4_, to accumulate, and consequently, to increase glycolytic intermediates, including glucose-6-PO_4_, leading to stimulation of the PPP. Thus, increased NADPH, rather than decreased protein *O*-linked glycosylation, may be the mechanism for decreased oxidative stress and NTDs caused by the *O*-GlcNAc transferase inhibitor.

We recognize that resistance of the B6 strain to diabetic embryopathy that is observed by us and by some other laboratories (personal communication) is not observed by some other groups that have used the C57Bl/6J or C57Bl/6CrJ strains in the study of diabetic embryopathy [35,36,37]. In the studies in which C57Bl/6J or C57Bl/6CrJ strains have been successfully employed, the subcutaneously-implanted insulin pellets that we found are essential to prevent severe hyperglycemia and pregnancy loss in the periimplantation period [13] either were not used, or were surgically removed after implantation, so the glucotoxicity may be more severe than that to which our mice are subjected [35,36,37]. There could be other procedural, dietary, or environmental variations in different laboratory settings that might influence penetrance of malformations in B6 diabetic pregnancies. Nonetheless, the same high glucose concentrations that induced oxidative stress, which has been shown to play a causal role diabetic embryopathy in several animal studies [7,8,9,10,11,12], in FVB LG-ESC, did not induce oxidative stress in B6 LG-ESC. This indicates that there are genetic background differences between the two mouse strains in their responsiveness to the elevated glucose concentrations that occur during maternal diabetes.

There are some limitations of this study that should be recognized. For example, we did not test whether stimulation of the PPP by GlcN, and consequently, increased reduction of GSH from GSSG, is inhibited by high glucose in FVB LG-ESC, although these experiments are currently underway in our laboratory. Although a cell culture model of embryonic neuroepithelium is valuable to study the biochemical and molecular processes that lead to NTDs in diabetic embryopathy, it does not model the physical processes associated with convergent extension that occur during neural tube closure which could be affected by maternal diabetes. On the other hand, we have shown that high glucose does not inhibit LG-ESC to form 3-D neural cysts, which have been shown by others to model neural tube formation [38,39], so this may not be a shortcoming of our 2-D cell culture model. This study only examined effects of GlcN utilization and high glucose-induced oxidative stress by ESC that give rise to neuronal precursors, but we did not examine whether these processes are affected in other cell types that are prone to malformation in diabetic pregnancy, such as cardiac progenitors. Finally, although study GlcN metabolism, incorporation, and oxidative stress in intact FVB and B6 embryos is desirable, it is not possible to lower or eliminate GlcN in vivo, as explained previously. However, metabolomic profiling using dense isotopologues of GlcN, glucose, and glutamine administered to the pregnant mothers may make it possible to obtain further information on the role of GlcN on diabetic embryopathy in vivo.

## 5. Conclusions

The data reported here suggest that differential effects of GlcN on G6PD, the rate-controlling enzyme of the PPP, and therefore, on production of NADPH, with its consequent regulation of antioxidant capacity, may be responsible for the differential susceptibility of FVB and B6 embryos to diabetic embryopathy. Further investigation is needed to better understand GlcN metabolism during normal and high glucose conditions on different genetic backgrounds, and whether any differential GlcN-dependent pathways might be amenable for therapeutic intervention.

## Data Availability

Data is contained within the article.
